# Carbohydrate Antigen 50: Values for Diagnosis and Prognostic Prediction of Intrahepatic Cholangiocarcinoma

**DOI:** 10.3390/medicina56110616

**Published:** 2020-11-16

**Authors:** Sukanya Luang, Karuntarat Teeravirote, Waraporn Saentaweesuk, Prasertsri Ma-In, Atit Silsirivanit

**Affiliations:** 1 Department of Biochemistry, Faculty of Medicine, Khon Kaen University, Khon Kaen 40002, Thailand; sukany@kku.ac.th (S.L.); teeravirotek@gmail.com (K.T.); prasertsri.main@gmail.com (P.M.-I.); 2 Cholangiocarcinoma Research Institute, Khon Kaen University, Khon Kaen 40002, Thailand; 3 Faculty of Pharmacy, Mahasarakham University, Maha Sarakham 44150, Thailand; waraporn.sa@msu.ac.th

**Keywords:** tumor marker, CA50, bile ducts, liver, glycan

## Abstract

*Background and objectives:* Cancer-associated carbohydrate antigen 50 (CA50) is a marker for detection of gastrointestinal cancers, especially of pancreatic and colon cancer. In this study, the power of CA50 as a diagnostic and prognostic marker was evaluated in intrahepatic cholangiocarcinoma (iCCA). *Materials and Methods:* Serum CA50 levels of iCCA patients and non-cholangiocarcinoma controls (non-CCA, including healthy persons and patients with benign biliary diseases and other gastrointestinal cancers) were measured using MAGLUMI^®^800 CLIA analyzer. Diagnostic and prognostic values of serum CA50 levels were evaluated. *Results:* CA50 levels in the sera of iCCA patients were significantly higher than those of non-CCA controls (*p* < 0.001, Mann–Whitney U test). Using cut-off value of 25 U/mL, CA50 provided 65.9% sensitivity, 87.3% specificity, and 80.1% accuracy for diagnosis of iCCA. Serum CA50 levels were increased and associated with the severity of bile duct pathology. In addition, a higher level of CA50 was associated with poor clinical outcome and shorter survival in iCCA patients. Multivariate survival analysis by Cox regression model revealed the potential of CA50 as an independent poor prognostic indicator for iCCA, regardless of the age, sex, histological types, or tumor stages. *Conclusions:* CA50 can be a diagnostic and poor prognostic marker candidate for iCCA.

## 1. Introduction

Cholangiocarcinoma (CCA) is one of the most common primary liver malignancies. It is classified into three types, intrahepatic, perihilar, and distal CCA, based on their anatomic location to help the therapeutic treatment plan [1]. CCA incidence and mortality is increasing worldwide due to delayed diagnosis and treatment because the disease is asymptomatic at the early stage. Therefore, several CCA markers, such as carbohydrate antigens, have been applied for diagnosis, prognostic stratification, and treatment prediction. As the clinically nonspecific marker, carbohydrate antigen 19-9 (CA19-9) and carcinoembryonic antigen (CEA) are either individually or combinedly used for CCA diagnosis and prognosis with variable sensitivity and specificity [2,3,4,5]. However, a continual discovery of potential tumor markers for CCA is still crucial for these purposes. 

Cancer-associated carbohydrate antigen 50 (CA50) is a ganglioside glycoprotein and is a biomarker for the diagnosis and prognosis of gastrointestinal malignancies, especially of pancreatic and colorectal cancers [6,7]. The expression level of CA50 has been measured for pancreatic and colorectal cancers and also for other cancers such as breast, lung, renal, prostatic, and ovarian cancers [6]. In addition, it can distinguish the patients of benign disease of atrophic gastritis, pancreatitis, ulcerative colitis, pneumonia, cirrhosis, and autoimmune diseases from healthy persons [8]. The monoclonal antibody that defines CA50 reacts with both the afucosyl form of sialylated Lewis A carbohydrate moiety and sialylated Lewis A moiety on the cancer cell surface [9]. CA50 epitope is assumed to be similar to CA19-9 epitope (sialyl-Lewis A). In fact, both CA19-9 and CA50 are valuable markers for the differentiation of pancreatic cancer from benign diseases of the pancreatobiliary tract [10,11]. However, in contrast to the limited expression of CA 19-9 in gastrointestinal malignancy, high CA50 levels can also be seen in malignant tumors outside the digestive tract. In spite of extensive study on the diagnostic value of CA50 for various malignancies, its expression level in the sera of CCA patients has not much been explored. The previous studies by Haglund et al. and Watanabe et al. demonstrated that CA50 was elevated in CCA tissues [12,13]. In our study, we have investigated the potential of CA50 to be a marker for diagnosis of CCA by measuring the CA50 level in serum from intrahepatic cholangiocarcinoma (iCCA) patients compared with non-CCA controls. In addition, the power of CA50 in prognosis of CCA was also demonstrated. 

## 2. Materials and Methods

### 2.1. Patients and Samples

Serum samples from healthy individuals (HE) were obtained from asymptomatic persons with a normal level of fasting blood sugar (<110 mg/dL), aspartate aminotransferase (AST, ≤35 U/mL), alanine aminotransferase (ALT, ≤37 U/mL), and alkaline phosphatase (ALP, < 121 U/mL) who visited Srinagarind Hospital, Khon Kaen University, Khon Kaen, Thailand, for their annual health check-up. The serum samples from benign biliary diseases (BBD, *n* = 23), other gastro-intestinal cancers (OCA, *n* = 33), and iCCA (*n* = 85) were obtained from the Specimen Bank of the Cholangiocarcinoma Research Institute, Khon Kaen University. The patients of BBD, OCA, and iCCA were definitely diagnosed by histopathological study of resected tumor tissues at the Department of Pathology, Srinagarind Hospital. The OCA group consisted of hepatoma and carcinoma of ampulla of Vater (*n* = 6), colon and colorectal cancer (*n* = 10), pancreatic cancer (*n* = 7), and gastric cancer (*n* = 10). The informed consents were obtained from each subject. The study protocol was approved by the Khon Kaen University Ethics Committee for Human Research based on the Declaration of Helsinki and the ICH Good Clinical Practice Guidelines (HE621176). 

### 2.2. CA50 and CA19-9 Analysis

The serum CA50 and CA19-9 levels were measured using an automatic chemiluminescence immunoassay (CLIA) on the MAGLUMI^®^800 CLIA analyzer from SNIBE Co., Ltd. (Shenzhen New Industries Biomedical Engineering Co., Ltd.), Shenzhen, China. 

### 2.3. Statistical Analysis

The statistical analyses were performed using IBM-SPSS statistics version 26 (IBM Corp., Armonk, NY, USA) and GraphPad-Prism version 8.4.2 (GraphPad Software, San Diego, CA, USA). The difference of CA50 among the groups of subjects was analyzed by Mann–Whitney U test. Univariate and multivariate Cox regression analyses were used to identify the factors that influence the survival of CCA patients. Kaplan–Meier plot and log-rank test were used to analyze the association of CA50 and median survival time of the patients. 

## 3. Results

### 3.1. CA50 Was Elevated in the Sera of CCA Patients and Has a Diagnostic Value for iCCA

**Figure 1:**
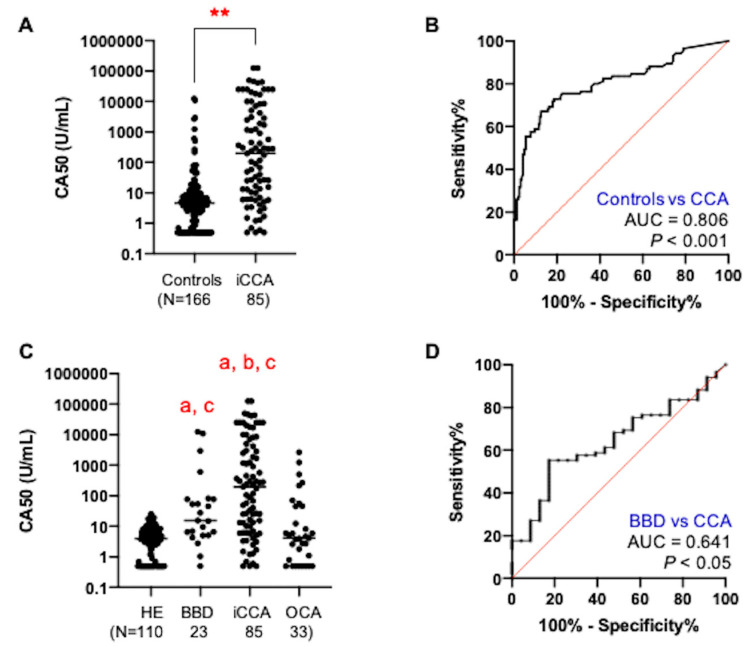
Serum CA50 level of iCCA patients and non-CCA control. (**A**) Scatter plot showed the level of CA50 in iCCA patients, compared with non-CCA control. (**B**) ROC analysis to analyze the power of CA50 in discriminating iCCA from non-CCA control. (**C**) Scatter plot showed the level of CA50 in healthy persons (HE), patients of benign biliary diseases (BBD), other gastrointestinal cancers (OCA) and iCCA. (**D**) Receiver operating characteristic (ROC) analysis to analyze the power of CA50 in discriminating iCCA from BBD cases. The significant difference of CA50 level among the cases is indicated by stars (**) for *p* < 0.001, while the small letter indicates *p* < 0.05 (a vs. HE, b vs. BBD, and c vs. OCA).

To investigate the potential of serum CA50 level for CCA diagnosis, we measured the expression level of CA50 in preoperative serum samples of iCCA patients (*n* = 85) and compared with those of 166 non-CCA controls, including 110 HE, 23 BBD, and 33 OCA (Table 1). Serum CA50 levels of iCCA patients varied, ranging from 0.5 to 125,000 U/mL with the median value of 197.3 U/mL, which is significantly higher than that of non-CCA controls (median = 4.6 U/mL) (Figure 1A, *p* < 0.001). Receiver operating characteristic (ROC) curve analysis revealed that CA50 could differentiate iCCA patients from non-CCA controls with the area under curve (AUC) of 0.806 (Figure 1B, *p* < 0.001). Using the cut-off value of 25 U/mL, CA50 was found to provide 65.9% sensitivity, 87.3% specificity, and 80.1% accuracy to discriminate iCCA patients from non-CCA controls (Table 2). The diagnostic values of CA50 were comparable with those of CA19-9, a standard marker for diagnosis of CCA. 

### 3.2. The Increase of Serum CA50 Was Associated with the Severity of Bile Duct Pathology

Comparing between iCCA and BBD, the level of CA50 in iCCA was significantly higher than that in BBD (Figure 1C, *p* < 0.05). The median values of serum CA50 level of both BBD and iCCA groups were significantly higher than that of OCA group (4.1 U/mL) (Figure 1C, *p* < 0.0001). Using the cut-off value of 25.0 U/mL, 47.8% (11/23) of BBD and 27.3% (9/33) of OCA cases were found to be positive for CA50 (Table 1). As shown in Figure 1D, ROC analysis revealed that CA50 can differentiate iCCA from BBD with an AUC of 0.641 (*p* < 0.05). Using Youden’s index calculated from ROC analysis [14], a new cut-off value of CA50 was determined to be 85.3 U/mL, which provided the specificity to exclude 82% (19/23) of BBD, 84% (28/33) of OCA, and 100% (110/110) of HE cases, although the sensitivity to detect iCCA was decreased to 55.3% (47/85), as shown in Table 2. 

### 3.3. High Level of CA50 of iCCA Patients Indicates Poor Prognosis and Shorter Survival

**Figure 2:**
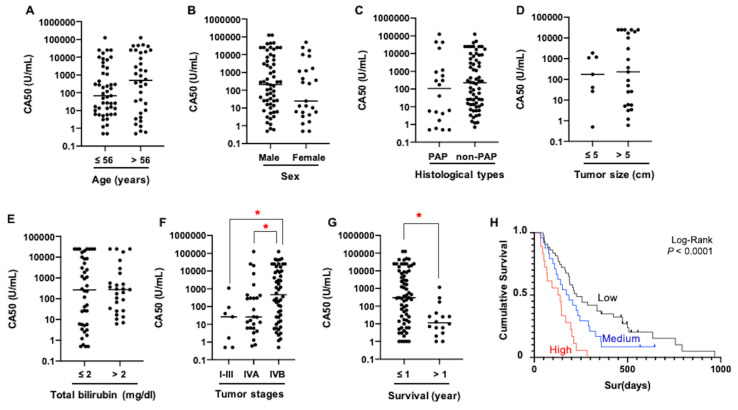
Correlation between CA50 level and clinicopathological data of iCCA patients. The level of CA50 was compared among the patients with different (**A**) age, (**B**) sex, (**C**) histological types, (**D**) tumor size, (**E**) total bilirubin, (**F**) tumor stages, and (**G**) 1 year survival; using Mann–Whitney U test (* *p* < 0.05). (**H**) Kaplan–Meier plot and log-rank test for survival analysis of CA50.

To investigate the potential of using CA50 as a prognostic marker for iCCA, the correlation between patient clinical outcomes and serum CA50 level was analyzed. Differences of CA50 level between iCCA subgroups were determined using Mann–Whitney U test (Figure 2). Serum CA50 level of the iCCA subgroup with tumor stage IVB and survival ≤1 year was significantly higher than that of the subgroup with stage I–III/IVA and survival >1 year (*p* < 0.05). However, serum CA50 level was not associated with age, sex, histopathological types, tumor size, and total bilirubin level of the patients. Then, iCCA patients were subcategorized according to the median (197.3 U/mL) and mean (8348.0 U/mL) values of serum CA50 levels into 1) Low (≤197.3 U/mL, *n* = 43), 2) Medium (197.4–8348.0 U/mL, *n* = 24), and 3) High (>8348.0 U/mL, *n* = 18) groups. Univariate Cox proportional hazard analysis revealed that the iCCA patients with high CA50 have 3.629 times higher hazard ratio compared with those with low CA50 (Table 3, *p* < 0.05). Multivariate survival analysis suggested the potential of using CA50 as an independent prognostic indicator for iCCA regardless of age, sex, histological type, and tumor stage of the patients (Table 3, *p* < 0.05). As shown in Figure 2H, Kaplan–Meier plot and log-rank analysis showed that the patients with a high level of CA50 had the shortest survival (127.0 days, 95% CI of 35.5–128.5 days), compared with those with medium CA50 (172.0 days, 95% CI of 91.6–252.4 days) and low CA50 (227.0 days, 95% CI of 161.5–292.5 days) (*p* < 0.001). The overall median survival of iCCA patients and 95% CI were 189.0 and 152.9–225.1 days, respectively. 

## 4. Discussion

Altered glycosylation is seen in CCA, and aberrant expression of glycans plays significant roles in CCA progression, leading to poor survival of patients. Beside involving in tumor progression [15,16,17,18,19], the CCA-associated glycans were found to be the potential biomarkers for diagnosis and prognostic prediction of the disease [18,20,21].

In this study, we have analyzed the serum level of sialyl-associated glycan antigen CA50 in iCCA, BBD, and healthy control groups. Our data showed that CA50 level was associated with the severity of bile duct pathology, as its level gradually increased in BBD and iCCA, suggesting the potential use of CA50 as the indicator of bile ducts pathology. The previous study by Haglund et al. and Watanabe et al. demonstrated the increasing of CA50 level in CCA tissues and showed it could be secreted from CCA cells, suggesting that CA50 is a tumor origin tumor marker [12,13]. Our present study was performed in a larger cohort of CCA (*n* = 85) and various non-CCA cases (*n* = 166). When we set the cut-off value at 25 U/mL, CA50 was able to differentiate iCCA patients from non-CCA control with 65.9% sensitivity, 87.3% specificity, and 80.1% accuracy. The diagnostic values provided by CA50 were slightly higher than that of a general serum marker for diagnosis of CCA, CA19-9 (65.9% sensitivity, 86.1% specificity, and 79.3% accuracy). Combined analysis of these markers may improve their power in diagnosis of CCA. 

When the cut-off value was set at 85.3 U/mL, the CA50 could exclude 82% (19/23) of BBD, 84% (28/33) of OCA, and 100% (110/110) of HE cases, suggesting that the patients with high CA50 have high possibility of having iCCA. Although CA50 was previously reported to increase in patients with gastrointestinal cancers, for example, gastric cancer and pancreatic cancer [8], our results demonstrated the degree of increase of CA50 in iCCA patients was significantly higher than that of OCA and other control groups. Related to this, Tao et al. previously demonstrated that the serum CA50 levels in CCA and hepatoma patients were comparable and statistically not different from each other [22]. This difference might be due to the different etiology of CCA in Thailand and other countries. The CCA in Thailand is almost exclusively associated with liver fluke, *Opisthorchis viverrini* infection, while the CCA in other countries is associated with various factors, for example, primary sclerosing cholangitis and primary biliary cirrhosis. Previous studies comparing the epigenetics and gene expression profile revealed that CCA with different etiology exhibits different gene expression patterns [23,24,25], therefore the increase of a particular molecule may not be observed and applicable for the diagnosis of all types of CCA. To improve the detection of CCA, combined analysis of multiple markers may possibly provide the better answer. 

In this study, serum CA50 level was correlated with the clinical outcomes of CCA patients. High level of CA50 in the serum was associated with the advanced tumor stage and shorter survival of iCCA patients. Previous studies on CA50 expression in gastric and pancreatic cancer patients showed that the high level of CA50 is significantly associated with shorter survival of the patients [26,27,28]. Previous studies by Juntavee et al. (2005) and Wattanavises et al. (2019) demonstrated the increase of sialylation, as represented by sialyl Lewis A antigen and MAL-II binding glycan, in CCA, compared with normal bile ducts and parenchymal cells, in association with poor prognosis and short survival of CCA patients [16,19]. These glycans are involved in metastasis and chemoresistance of CCA cells [16,19]. Thus, CA50, another sialylated glycan, may also play roles in CCA metastasis and chemoresistance, resulting in poor prognosis.

## 5. Conclusions

CA50 could be a candidate marker for CCA diagnosis and prognostic prediction, as the higher level of CA50 was associated with advanced bile duct pathology and shorter survival of iCCA patients. Further analysis using a combination of CA50 and other CCA markers may enhance the sensitivity and specificity in diagnosis of CCA, resulting in better outcomes of CCA treatments.

## Figures and Tables

**Table 1:** Serum level of carbohydrate antigen CA50 in non-cholangiocarcinoma (non-CCA) and intrahepatic cholangiocarcinoma (iCCA) patients.

Index	Group of Subjects			
	Healthy (HE)	Benign (BBD)	Other Cancers (OCA)	iCCA
*n*	110	23	33	85
**CA50 Level (U/mL)**				
Mean	5.1	1195.0	156.9	8348.0
Median	4.0	15.5	4.1	197.3
Min	0.5	0.5	0.5	0.5
Max	25.6	12,460.0	2664.0	125,000.0
**Number of Cases at Cut-Off CA50 = 25.0 U/mL**				
CA50 ≤ 25.0 U/ml	109	12	24	29
CA50 > 25.0 U/ml	1	11	9	56
**Number of Cases at Cut-Off CA50 = 85.3 U/mL**				
CA50 ≤ 85.3 U/ml	110	19	28	38
CA50 > 85.3 U/ml	0	4	5	47

**Table 2:** Diagnostic values of CA50 and carbohydrate antigen 19-9 (CA19-9).

Analyses	Non-CCA vs. iCCA		BBD vs. iCCA
	CA19-9	CA50	CA50
**Mann–Whitney U test**			
*p*	<0.001	<0.001	0.039
**ROC Analysis**			
AUC	0.797	0.806	0.641
*p*	<0.001	<0.001	0.039
Cut-off value	37 U/mL	25 U/mL	85.3 U/mL
Sensitivity (%)	65.9	65.9	55.3
Specificity (%)	86.1	87.3	82.6
Positive predictive value (%)	70.9	72.7	92.2
Negative predictive value (%)	83.1	83.3	33.3
False positive (%)	13.9	12.7	17.4
False negative (%)	34.1	34.1	44.7
Accuracy (%)	79.3	80.1	61.1

**Table 3:** Univariate and multivariate Cox regression analyses.

Parameters	*n*	Univariate Analysis			Multivariate Analysis		
		HR	95% CI	*p*	HR	95% CI	*p*
**Age (years)**							
≤56	49	1	-		1	-	
>56	36	1.236	0.777–1.965	0.370	1.506	0.908–2.500	0.113
**Sex**							
Male	60	1	-		1	-	
Female	25	0.729	0.444–1.199	0.213	0.725	0.425–1.235	0.236
**Histological types**							
Papillary	20	1			1	-	
Non-papillary	65	1.903	1.060–3.418	0.031	1.963	1.036–3.720	0.039
**Tumor stage**							
I–III	7	1	-			-	
IVA	25	1.954	0.783–4.881	0.151	1.801	0.691–4.696	0.229
IVB	52	2.725	1.129–6.577	0.026	1.744	0.676–4.498	0.250
**CA50 (U/mL)**							
Low	43	1	-			-	
Medium	24	1.659	0.964–2.854	0.068	1.447	0.689–3.040	0.329
High	46	3.629	1.961–6.714	<0.001	2.988	1.269–7.032	0.012
